# Biodistribution, pharmacokinetics, and organ-level dosimetry for ^188^Re-AHDD-Lipiodol radioembolization based on quantitative post-treatment SPECT/CT scans

**DOI:** 10.1186/s40658-018-0227-6

**Published:** 2018-12-07

**Authors:** Pedro L. Esquinas, Ajit Shinto, Koramadai K. Kamaleshwaran, Jephy Joseph, Anna Celler

**Affiliations:** 1 Department of Radiology, University of British Columbia, Vancouver, British Columbia Canada; 2 Medical Imaging Research Group, Vancouver, British Columbia Canada; 3 Department of Nuclear Medicine, Kovai Medical Center and Hospital, Coimbatore, Tamil Nadu India

**Keywords:** Rhenium-188, AHDD-Lipiodol, Quantitative SPECT, Radioembolization, Dosimetry, Hepatocellular carcinoma

## Abstract

**Background:**

Rhenium-188-labelled-Lipiodol radioembolization is a safe and cost-effective treatment for primary liver cancer. In order to determine correlations between treatment doses and patient response to therapy, accurate patient-specific dosimetry is required. Up to date, the reported dosimetry of ^188^Re-Lipiodol has been based on whole-body (WB) planar imaging only, which has limited quantitative accuracy. The aim of the present study is to determine the in vivo pharmacokinetics, bio-distribution, and organ-level dosimetry of ^188^Re-AHDD-Lipiodol radioembolization using a combination of post-treatment planar and quantitative SPECT/CT images. Furthermore, based on the analysis of the pharmacokinetic data, a practical and relatively simple imaging and dosimetry method that could be implemented in clinics for ^188^Re-AHDD-Lipiodol radioembolization is proposed.

Thirteen patients with histologically proven hepatocellular carcinoma underwent ^188^Re-AHDD-Lipiodol radioembolization. A series of 2–3 WB planar images and one SPECT/CT scan were acquired over 48 h after the treatment. The time-integrated activity coefficients (TIACs, also known as residence-times) and absorbed doses of tumors and organs at risk (OARs) were determined using a hybrid WB/SPECT imaging method.

**Results:**

Whole-body imaging showed that ^188^Re-AHDD-Lipiodol accumulated mostly in the tumor and liver tissue but a non-negligible amount of the pharmaceutical was also observed in the stomach, lungs, salivary glands, spleen, kidneys, and urinary bladder. On average, the measured effective half-life of ^188^Re-AHDD-Lipiodol was 12.5 ± 1.9 h in tumor. The effective half-life in the liver and lungs (the two organs at risk) was 12.6 ± 1.7 h and 12.0 ± 1.9 h, respectively. The presence of ^188^Re in other organs was probably due to the chemical separation and subsequent release of the free radionuclide from Lipiodol.

The average doses per injected activity in the tumor, liver, and lungs were 23.5 ± 40.8 mGy/MBq, 2.12 ± 1.78 mGy/MBq, and 0.11 ± 0.05 mGy/MBq, respectively. The proposed imaging and dosimetry method, consisting of a single SPECT/CT for activity determination followed by ^188^Re-AHDD-Lipiodol clearance with the liver effective half-life of 12.6 h, resulted in TIACs estimates (and hence, doses) mostly within ± 20% from the reference TIACs (estimated using three WB images and one SPECT/CT).

**Conclusions:**

The large inter-patient variability of the absorbed doses in tumors and normal tissue in ^188^Re-HDD-Lipiodol radioembolization patients emphasizes the importance of patient-specific dosimetry calculations based on quantitative post-treatment SPECT/CT imaging.

**Electronic supplementary material:**

The online version of this article (10.1186/s40658-018-0227-6) contains supplementary material, which is available to authorized users.

## Background

Liver cancer is one of the leading causes of cancer-related deaths worldwide [1]. For patients suffering from primary or secondary liver cancer, resection represents the therapy of choice, but only a minority of patients fulfill the criteria for resection surgery or liver transplantation [2]. When surgery is not an option, other treatment strategies are available such as systemic chemotherapy for liver metastases [3], hepatic arterial embolization (with or without chemotherapy), radiofrequency ablation [4], or brachytherapy [5]. External beam radiotherapy might also be effective but can only be applied in localized diseases. Another possible treatment for these patients is trans-arterial radioembolization.

The majority of radioembolization procedures involve the use of microspheres (glass or resin) labelled with ^90^Y [6]. The high-energy electrons emitted by ^90^Y during its *β*-decay (*E*_max_ = 2.2 MeV [7]) make this radionuclide suitable for treating tumors. However, since ^90^Y is a pure *β*-emitter, imaging the microsphere biodistribution after the treatment is very difficult. It is commonly performed using Bremsstrahlung SPECT [8], which yields very poor quality images and requires sophisticated techniques for quantitative measurements [9, 10].

Alternatively, quantitative ^90^Y imaging can be performed using PET/CT [11, 12], but this approach also presents challenges. Due to the very low emission yield of positrons by ^90^Y, these PET images suffer from high levels of noise and careful optimization of the reconstruction parameters is required to achieve low quantification errors in phantom studies [12]. Furthermore, PET/CT scanners are typically less available than SPECT/CT cameras in nuclear medicine departments. Additionally, the commercially available ^90^Y microspheres themselves are expensive, making this treatment option inaccessible for many patients. The situation is especially difficult in the developing countries where hepatocellular carcinoma has high prevalence in the population [13].

An alternative to ^90^Y is the use of ^131^I (8.02 days), which emits high-energy electrons (*E*_max_ = 0.806 MeV [14]) and 364-keV photons, suitable for SPECT imaging. Compared with surgery alone, the use of ^131^I-labelled-Lipiodol (in an adjuvant setting) to treat hepatocellular carcinoma has been effective in improving recurrence-free and overall survival of patients. Additionally, in a palliative setting, ^131^I-Lipiodol has improved survival of patients with portal thrombosis [15]. Although image-based dosimetry of ^131^I-Lipiodol radioembolization is scarce, a clinical study by Becker et al*.* [16] demonstrated the correlation between image-based tumor dosimetry and the therapeutic response. Overall, the authors showed that tumor doses above 280 Gy resulted in 84% of patients responding after the first ^131^I-Lipiodol treatment, and no responses were recorded below this threshold. It is important to highlight that dosimetry calculations in this study were performed using on quantitative ^131^I SPECT images with corrections for attenuation, scatter, and dead-time.

Despite these encouraging results, one of the major limitations of ^131^I-Lipiodol radioembolization is that, due to ^131^I long half-life and high-energy photon emissions, it requires long hospitalization times and, in some cases, limited administered activities to minimize toxicity [17]. Additionally, the maximum electron energy of 606 keV is not sufficient to treat medium to large tumors effectively.

To remedy these problems, the use of ^188^Re has been proposed (*E*_max_ = 2.1 MeV, half-life = 17 h [18]). Not only ^188^Re emits electrons with energies similar to those of ^90^Y, thus potentially having similar therapeutic effects, but also photons which are suitable for imaging. As demonstrated in our previous work [19, 20], ^188^Re 155 keV *γ*-emissions allow us to perform quantitative ^188^Re SPECT imaging which can be used for accurate image-based dosimetry. In addition, the availability of ^188^Re through the ^188^W/^188^Re generator [21–23] makes the treatment less costly and more practical than ^90^Y, because ^188^Re can be produced on site in the nuclear medicine department.

The main clinical experience with ^188^Re radioembolization comes from the study performed in the scope of an international collaboration sponsored by the International Atomic Energy Agency (IAEA) that involved eight countries [13, 24]. In this study, 185 patients received radioembolization using ^188^Re-labelled 4-hexadecyl-1,2,9,9-tetramethyl-4,7-diaza-1,10-decanethiol-Lipiodol (^188^Re-HDD-Lipiodol [25]). The results indicated that 25% of treated patients showed objective response (tumor size reduction by 50%, as seen on CT); stable disease was observed in 53% and tumor progression in 22%. Overall, this multi-center study showed that ^188^Re-HDD-Lipiodol is a safe and cost-effective treatment for primary liver cancer.

The organs at risk during radioembolization using ^188^Re HDD-Lipiodol are the lungs, liver, and bone marrow with maximum tolerated doses of 12 Gy, 30 Gy, and 1.5 Gy, respectively [26–28]. Zanzonico and Divgi [29] developed a clinical algorithm to estimate the patient-specific therapeutic activity for ^188^Re-HDD-Lipiodol radioembolization, which was used in the IAEA study. In this protocol, a pre-treatment dosimetry was performed based on the ^188^Re “scout” whole-body (WB) planar scan. This dosimetry was then used to estimate the maximum injected activity (i.e., the therapeutic dose) that would result in organs-at-risk doses below the tolerance limits. This protocol relied on the assumption that biological clearance of ^188^Re-HDD-Lipiodol by tumor and other organs was very slow, and therefore, the pharmacokinetics of the radiotracer were modeled as a mono-exponential function with an effective half-life equal to ^188^Re physical half-life (17 h).

The in vivo biodistribution and organ dosimetry of ^188^Re-HDD-Lipiodol using post-treatment WB imaging were first investigated in a phase I clinical trial by Lambert et al. 2005 [30]. The authors of this study reported that, in fact, there was biological clearance of ^188^Re-HDD-Lipiodol from tissues. In this clinical trial, however, no quantitative SPECT/CT acquisitions were performed, and the reported radiation doses were derived from planar WB images only.

The main objective of the present work is to determine the in vivo pharmacokinetics and patient-specific dosimetry of ^188^Re-Lipiodol radioembolization using quantitative post-treatment imaging. As a second goal, the effect of the frequency and the timing of the post-treatment imaging on the calculated tumor/organs time-integrated activity coefficients (TIACs) (a quantity proportional to the absorbed dose) are investigated. The aim of this second objective is to establish a practical and relatively simple method that could be implemented in clinics and which would allow for a routine determination of doses delivered in ^188^Re-HDD-Lipiodol radioembolization procedures.

## Methods

### Preparation of ^188^Re-AHDD-Lipiodol

Rhenium-188, in the form of sodium perrhenate (Na^188^ReO_4_), was eluted from the ^188^W/^188^Re generator (iTG–Isotopen Technologien München, Germany) using a 0.9% sodium-chloride (NaCl) solution. Rhenium-188 Lipiodol labelling was performed using diacetylated 4-hexadecyl-1,2,9,9-tetramethyl-4,7-diaza-1,10-decanethiol-Lipiodol (^188^Re-AHDD-Lipiodol) labelling kits, following the procedure described previously by Lee et al. 2007 [31]. The AHDD labelling kits were obtained from Seoul National University Hospital (Seoul, South Korea). Routine quality control testing for radiochemical purity was done prior to every patient injection, consisting mainly of thin layer chromatography.

The activity of ^188^Re-AHDD was measured before and after the addition of Lipiodol using a Capintec CRC-25 R dose calibrator (Capintec, USA). Following previous recommendations [32, 33], the dose-calibrator dial-setting for ^188^Re was set to 621 × 10. It was noted that, after mixing Lipiodol with ^188^Re-AHDD, the dose-calibrator reading of activity decreased by 12–15% due to the attenuation of the ^188^Re photon flux caused by the presence of Lipiodol (density 1.28 g/cm^3^) in the sample. The vial was subsequently mixed in a vortex mixer and kept in a centrifuge for 15 min at 3000 rpm. The supernatant was separated from the mixture using a long bore lumbar puncture needle. Although the radioactivity in Lipiodol phase was found to be 60–70% of the original activity, the actual product that was drawn into the syringe to be administered to a patient contained only 50–60% of this activity. This was due to the inevitable loss of Lipiodol phase sticking to the inner wall of the glass vial, due to its adhesive property.

Overall, the estimated relative uncertainty of the administered activity was 10.5%. We estimated this uncertainty by adding in quadrature the following uncertainties: (1) the relative uncertainty of the ^188^Re activity reading prior to addition of Lipiodol (0.5%, as reported in Zimmerman et al. 1999 [32]), (2) the variability of the activity reading due to the addition of Lipiodol to the original sample (3%), and (3) the variability of the residual activity left in the vial (10%).

### Patient selection criteria

Patients with histologically proven hepatocellular carcinoma who fell under Child-Pugh class A with adequate bone marrow and liver function were selected for this retrospective study. A total of 24 patients were screened for the study and those who demonstrated bi-lobular involvement in contrast-enhanced CT (ceCT) images were accepted. Exclusion criteria were as follows: eligibility for liver resection, pregnancy, and breast feeding, age below 18 years old, Child-Pugh C, poor general condition (Karnofsky score below 70%), white blood cell count below 1500/*μ*L, and potentially toxic anticancer treatment in the preceding 6 weeks. Patients with tumors larger than 500 mL, or tumors involving both liver lobes were also not included. To comply with the radiation protection guidelines, inability to care for oneself and incontinence were additional contraindications. A written informed consent was obtained from all the patients before them being enrolled in the study.

A total of 13 patients (2 females, 11 males) with a mean age of 57 years (range 20 years to 72 years) were treated with ^188^Re-AHDD-Lipiodol radioembolization. In this selected group of patients, 8 patients suffered from cirrhosis and 4 patients suffered from portal vein thrombosis. Prior to ^188^Re-radioembolization, some patients received the following treatments: trans-arterial chemoembolization (two patients), radiofrequency ablation (one patient), and sorafenib (eight patients, but the treatment stopped 4 weeks prior to the ^188^Re-AHDD-Lipiodol radioembolization therapy).

### Treatment planning

The therapeutic activity of ^188^Re-AHDD-Lipiodol was calculated empirically based on the patient’s tumor volume and the maximum volume of administrable Lipiodol, which were determined using diagnostic ceCT imaging. Approximately, the target ^188^Re activity was 37 MBq (1 mCi) per milliliter of tumor. Such activity was considered to be the most feasible regimen for the following reasons. Firstly, each tumor can only accept a limited volume of radiolabelled Lipiodol (based on vascularity). Secondly, the AHDD labelling kits had approximately 50–60% extraction efficiency up to 3 mL of eluate. Therefore, only one kit could be used for smaller tumors and two kits could be used for large tumors, resulting in a final ^188^Re-labelled product volume ranging from 3 to 6 mL of Lipiodol. For very small tumors, the dose per unit volume was increased up to 92.5 MBq/mL (2.5 mCi/mL) whereas for very large tumors, the dose was decreased to approximately 14.8 MBq/mL (0.4 mCi/mL). Overall, the total administered activities ranged from 1296 to 7162 MBq. These activities were within the amounts administered in the IAEA study, which resulted in good patient tolerance to the treatment [13].

### Interventional radiology procedure and administration

The angiographic approach to administer ^188^Re-AHDD-Lipiodol was based on a planning triple-phase ceCT of the hepatic arterial system with a special emphasis on the tumor feeding vessels. Based on the patient anatomy and vasculature, either a Yashiro catheter (Temuro Corporation, Japan) or a Sim-1 French catheter (Johnson and Johnson, USA) were used to catheterize the celiac axis and the hepatic artery. If multiple vessels arising from the hepatic artery were supplying the tumor, then a 5-French sheath was placed in the hepatic artery to allow easy cannulation of each of the tumor vessels.

The pre-calculated activity and specified volume of radiolabelled Lipiodol was injected until a complete opacification of the tumor was achieved. If the dyna-CT showed partial opacification of the tumor, other feeding branches were selected and the same procedure was repeated until the whole tumor was covered. Finally, the tumor feeding vessels were embolized with gel-foam scrapings unless there was an associated portal vein occlusion in the patient. No pulmonary shunt assessment or coiling of any other arteries was done.

### Post-treatment image-based dosimetry

#### General methodology: the hybrid planar/SPECT method

**Fig. 1:**
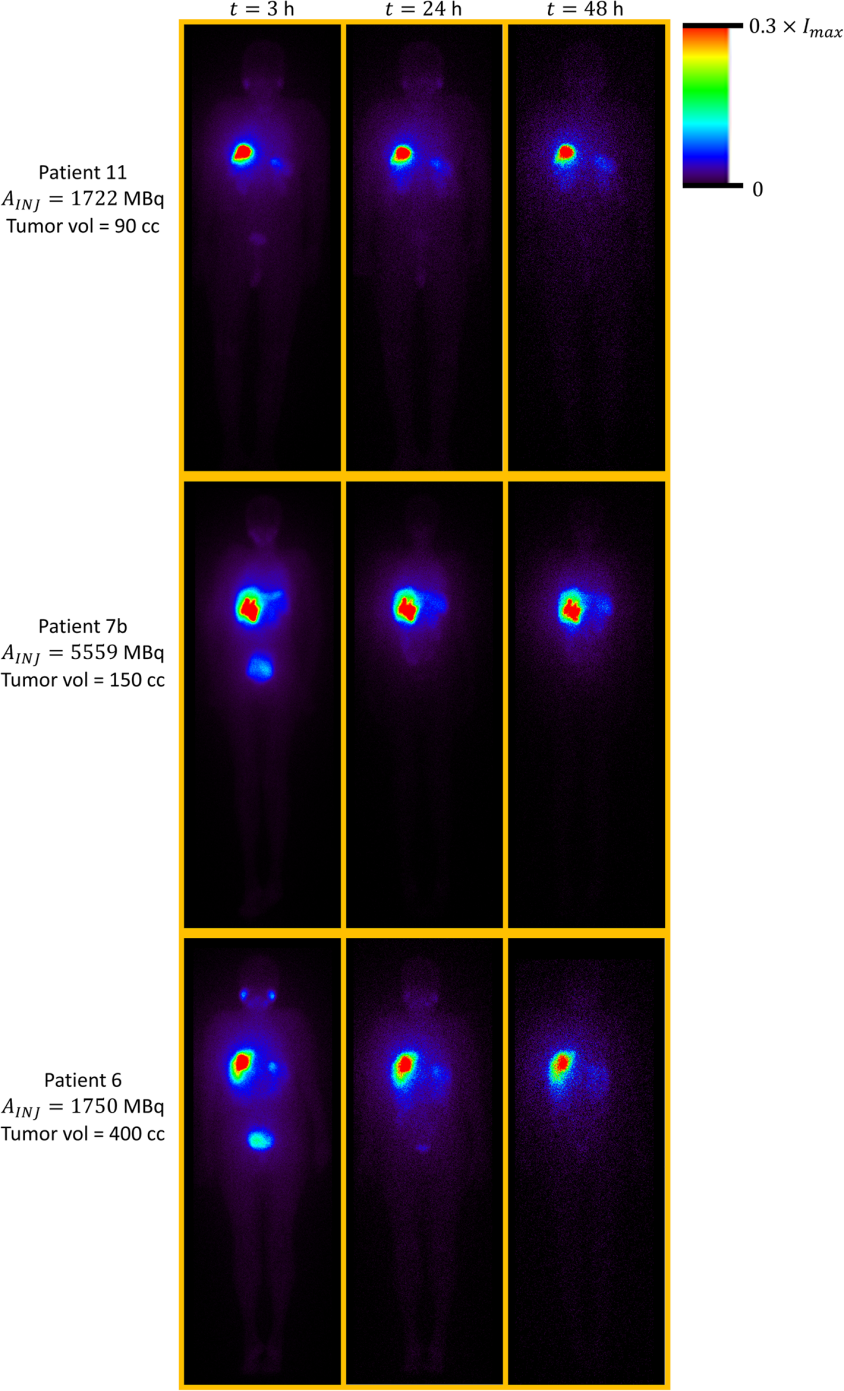
Whole-body planar images (anterior views) of three patients (no. 11, 7b, and 6) who received an intra-hepatic injection of ^188^Re-HDD-Lipiodol. The images were acquired at 3 h, 24 h, and 48 h post-administration of the radiotracer. The quantity *I*_*max*_ represents the maximum pixel intensity of each planar image

The goal of image-based personalized dosimetry calculations is to determine for each particular patient the dose (or dose-distribution) absorbed by his/her tumor(s) and organs at risk. For ^188^Re-AHDD-Lipiodol radioembolization, the organs at risk include the normal liver, lungs and bone marrow. In addition, other organs that might uptake ^188^Re-AHDD-Lipiodol are the stomach, kidneys, spleen, thyroid and salivary glands (see Fig. 1).

In order to calculate the absorbed dose, the hybrid planar/SPECT imaging protocol was applied in our study [34, 35]. It consisted of 2–3 WB planar scans at *t*_1_ = 3 ± 1 h, *t*_2_ = 24 ± 1 h, and *t*_3_ = 48 ± 4 h post-administration of ^188^Re-AHDD-Lipiodol and one SPECT/CT of the thorax/abdominal area at *t*_1_ = 3 ± 1 h. The WB images were used to determine the relative change of activity over time in regions of interest (i.e., the shape of the time-activity curves; TACs) while, the single SPECT/CT scan was used to determine the absolute TACs.

Secondly, the absolute TACs must be integrated over time to calculate the time-integrated activity (or total number of decays of ^188^Re) for each tumor and organ at risk. In our study, since only 2–3 data-points were available, the time-activity data were fit to mono-exponential functions. Details of the fit and the integration of TACs are described in the following sections.

Third, the time-integrated activity of each region of interest was divided by the injected activity to yield the TIACs. This quantity allowed us to compare the accumulation of ^188^Re-AHDD-Lipiodol in tumor/organs between different patients.

Finally, the TIACs were multiplied by the tumor and organ S-factors to determine the average absorbed dose (or dose per injected activity) in regions of interest.

A SymbiaT SPECT/CT camera (Siemens Medical, Germany) equipped with a high-energy collimator was used in all the scans. All the image-processing tasks described in this section were performed using QDOSE, the software for dosimetry calculations in radionuclide therapies (ABX-CRO, Germany). The dose calculations were performed with OLINDA/EXM 1.1 [36].

#### Whole-body imaging for relative TACs

In order to acquire the planar image of the whole-body, the speed of the patient-bed was set to 15 cm/min. The WB data at both anterior and posterior views were collected using a photopeak window centered at 155 keV (20% width) and were processed according to the MIRD pamphlet No. 16 [37]. Since no transmission scans were available, attenuation and/or scatter corrections were not applied to these images. For this reason, the 2-dimensional planar data were only used to determine the relative TACs.

In order to determine the time-activity curves of the source region *r*_*S*_, rough boundaries around these regions (organs/tumor) were first delineated on the WB planar images corresponding to the first time-point *t*_1_. This was done in order to separate these organs and tumors from the other regions containing activity. Subsequently, within these rough boundaries, a 40% threshold was applied to determine the count-rate within each segmented region of interest (ROI) in both the anterior and posterior views of the patient and the geometric mean was calculated. Then, the WB planar images acquired at *t*_2_ and *t*_3_ were co-registered to the first WB image at *t*_1_ using a rigid-registration algorithm and the segmented ROIs from the first image were transferred to the WB images that were acquired at the second and third time-points. As a result, the count-rate as a function of time was obtained for each source region *r*_*S*_. Since the count-rate is proportional to the activity for a given patient, we can assume that the relative change in count-rate equals the relative change of activity over time in the absence of dead-time losses. The count-rate losses in the WB images due to dead-time were corrected using our previously reported method [19]. For each source region, the relative time-activity data (*A*_*rel*_(*r*_*S*_, *t*)) were fit to a mono-exponential function using the weighted least-squares method:

1 $$ {A}_{\mathrm{rel}}\left({r}_S,t\right)=a\times {e}^{-\frac{\ln (2)}{T_{\mathrm{eff}}}t}, $$

where *a* and *T*_eff_ are the parameters of the fit. The quantity *T*_eff_ represents the effective half-life of ^188^Re-AHDD-Lipiodol in the source region *r*_*S*_. It is related to the physical half-life of ^188^Re (*T*_phys_ = 17 h) and the biological half-life of AHDD-Lipiodol (*T*_bio_) by the following formula:

2 $$ \frac{1}{T_{\mathrm{eff}}}=\frac{1}{T_{\mathrm{Phys}}}+\frac{1}{T_{\mathrm{bio}}}. $$

#### Quantitative SPECT for absolute TACs

During SPECT acquisitions, a total of 32 projections (20 s/view, 16 projections/detector) were acquired over 360° around the patient (angular step = 360/32 = 11.25°). The detector field-of-view covered the lungs and liver region in one bed position. The tomographic data were collected using a photopeak window centered at 155 keV (20% width) and two narrow secondary windows placed below and above the photopeak (5% width), respectively, to be used for triple-energy window (TEW) scatter correction. In addition, a low-dose CT (kVp = 130 kV, Exposure = 47 mAs) was acquired to be used for attenuation correction. The details of each patient’s protocol (patient information, administered activity, tumor volume, and imaging times) are reported in Table 1. Please note that for 4 patients (out of 13 patients) the imaging data were acquired at only two time-points (at *t*_1_ and *t*_2_).

**Table 1:** Patient information. The reported administered activities correspond to the dose-calibrator readings corrected for Lipiodol attenuation. The liver volumes were derived from pre-treatment contrast-enhanced CT images except for patients 3 and 8, which were derived from post-treatment SPECT/CT images

Patient no.	Age [year]	Sex	Injected Act. [MBq]	Imaging time post-injection [h]			Tumor volume [cc]	Liver volume [cc]
				*t*_1_	*t*_2_	*t*_3_		
1*	64	M	3405	2.7	26.3	50.3	13	1012
2*	69	M	6013	3.6	27.8	N/A	240	1200
3*	33	M	7162	5.2	27.5	52.6	1000	4030
4	59	M	1296	1.3	27.7	N/A	22	1194
5	71	M	1527	4.6	27.3	N/A	10	975
6	46	F	1750	3.1	24.7	49.2	400	1300
7a	20	F	5239	4.2	29.1	N/A	600	1400
7b	20	F	5559	3.6	28.2	51.3	150	1400
8*	51	M	2479	3.0	26.6	42.8	11	1260
9	53	M	2444	1.6	25.4	47.0	65	1300
10	72	M	2284	5.5	26.5	56.1	10	1300
11	68	M	1722	2.6	27.0	51.4	90	1152
12	62	M	2602	3.8	27.4	49.8	340	1200
13	71	M	1637	3.8	24.9	44.7	2.3	800

*Tomographic data in these patients were acquired without scatter windows. In these cases, the scatter was compensated using the attenuation map with broad-beam attenuation coefficients

Additionally, since the projection data for four patients’ tomographic studies (no. 1, 2, 3, and 8) did not include scatter windows, in these cases, the scatter correction was performed using the approximated approach with broad-beam attenuation coefficients. Also please note that one of the patients (patient no. 7) was treated twice.

The patient’s tomographic data were reconstructed using our in-house MIRG Software for quantitative ^188^Re SPECT, as described in our previous publication [19]. The protocol consisted of standard ordered-subset expectation maximization (OSEM [38]) reconstruction (8 subsets, 12 iterations) with corrections for attenuation (CT-based), scatter (TEW [39]), dead-time (based on the correction curve determined using phantom experiments [19, 40]), and resolution recovery [41]. The estimated dead-time losses in the patient projection data were always less than 6%.

The counts in the reconstructed images were converted into units of activity by applying a camera calibration factor determined from a planar scan of a point-source, following a procedure previously described [19].

To determine the absolute TACs (*A*(*r*_*S*_, *t*)), the source’s absolute activities were determined from the SPECT images (*A*(*r*_*S*_, *t*_1_)) and the relative TACs (*A*_*rel*_(*r*_*S*_, *t*)) were re-scaled according to these absolute source activities:

3 $$ A\left({r}_S,t\right)=a\times \frac{A\left({r}_S,{t}_1\right)}{A_{\mathrm{rel}}\left({r}_S,{t}_1\right)}{e}^{-\frac{\ln (2)}{T_{\mathrm{eff}}}t}, $$

where the parameters *a* and *T*_eff_ are from Eq. 1, and the quantity *A*_rel_(*r*_*S*_, *t*_1_) is obtained by evaluating Eq. 1 at *t*_1_.

In order to calculate the absolute activity in the organs of interest, their boundaries were manually delineated based on their physical size from CT images. This segmented CT-volume was applied to the SPECT image and the activity within the volume was integrated. The boundaries of tumors, however, were obtained by a different method. Firstly, a rough boundary was manually drawn (on the CT) covering the liver segment or lobe that contained the tumor (as reported by the physicians). Secondly, a fixed threshold was applied within this boundary region on the SPECT image such that the recovered volume would equal the reported tumor volume of Table 1.

#### Time-integrated activity, time-integrated activity coefficients, and organ-level dosimetry

The last step to determine the time-integrated activity $\overset{\sim }{A}\left({r}_S,{T}_D\right)$ in the source region *r*_*S*_ involved the following calculation:

4 $$ \overset{\sim }{A}\left({r}_S,{T}_D\right)={\int}_0^{{\mathrm{T}}_{\mathrm{D}}}A\left({r}_S,t\right) dt, $$

where *T*_*D*_ is the dose-integration period, which was equal to infinity in our study. The following assumptions were made about the TAC in time-intervals where no imaging data were available: for *t* = 0 to *t*_1_, the source-organ activity was assumed to grow linearly from 0 to *A*(*r*_*S*_, *t*_1_), and for *t* > *t*_3_, the activity was extrapolated assuming a mono-exponential clearance following the ^188^Re physical decay. The time-integrated activity $\overset{\sim }{A}\left({r}_S,{T}_D\right)$ was then calculated by analytical integration of this TAC. The resulting time-integrated activity in each source organ was divided by the injected activity (*A*_0_) to determine the TIAC $\overset{\sim }{a}\left({r}_S,{T}_D\right)$:

5 $$ \overset{\sim }{a}\left({r}_S,{T}_D\right)=\frac{\overset{\sim }{A}\left({r}_S,{T}_D\right)}{A_0}. $$

The absorbed radiation doses in organs of interest were calculated using OLINDA by combining the estimated TIACs with the pre-calculated organ-level S-factors of an adult male/female human phantom. The S-factors for each organ were adjusted to the organ mass of each patient obtained from CT images. The doses absorbed by tumors were obtained by multiplying the tumors’ TIACs by the ‘spherical model’ S-factors (adjusted for each tumor volume) available in OLINDA. Information and tabulated S-values for organs at risk and spheres of different sizes is included in the Additional file 1.

In this work, we determined pharmacokinetics and dosimetry for the regions which were visible within the field-of-view of SPECT/CT images, as only for them the activity could be accurately quantified. These regions included the tumor, entire liver, lungs, stomach, spleen, and kidneys. Please note that the “remainder of the body” TIAC term in OLINDA, which mostly corresponded to activity in salivary/parotid glands, thyroid, and urinary bladder (see the “Biodistribution of 188Re-AHDD-Lipiodol” section) was not determined in our study. The dose to bone marrow was not estimated due to the limited accuracy of image-based methods to determine it. An alternative method to estimate bone marrow dose in ^188^Re-Lipiodol studies based on blood-sample counting was described in Zanzonico and Divgi 2008 [29].

### Effect of timing and number of scans on the time-integrated activity coefficient

In order to develop a simple dosimetry protocol that could reduce the number of imaging time-points while maintaining the accuracy of dose calculations, we investigated the variability of the calculated pharmacokinetics and the TIACs (which are proportional to the radiation absorbed dose) as a function of the number and timing of image acquisitions. To do this, the time-activity data for each of the 10 patients that were scanned three times at *t* = {3 h, 24 h and 48 h} (Table 1) were divided into three subsets: {3 h, 24 h}, {3 h, 48 h}, and {24 h, 48 h}. The pharmacokinetics (i.e., effective half-lives) in tumor and organs at risk derived from each of these three subsets were compared to those obtained using all three data-points, considered here as the reference. Similarly, the TIACs calculated using each subset *X* of imaging data (${\overset{\sim }{a}}_X\left({r}_S,{T}_D\right)$) for the tumor, liver, and lungs (the main regions of interest for this therapy) were compared to the reference TIACs ($\overset{\sim }{a_{ref}}\left({r}_S,{T}_D\right)\Big)$, where *ref* = {3h, 24h, 48h} by calculating the percent difference:

6 $$ {\%}_{\mathrm{diff}}=\frac{\overset{\sim }{a_X}\left({r}_S,{T}_D\right)-\overset{\sim }{a_{ref}}\left({r}_S,{T}_D\right)}{\overset{\sim }{a_{ref}}\left({r}_S,{T}_D\right)}\times 100 $$

In addition, the TIACs of tumor, liver, and lungs were estimated for the following two cases: (a) using the activity obtained from the SPECT image at *t*_1_= 3 h and assuming that TAC shape is described by the physical decay of ^188^Re only (this method is referred as to ‘{3 h}+*T*_*phys*_’) and (b) using the activity obtained from the SPECT image at *t*_1_ = 3 h and assuming a mono-exponential decay with the average effective half-life of ^188^Re-AHDD-Lipiodol in liver tissue derived from the complete imaging dataset of patients (this method is referred as to ‘{3 h}+*T*_eff_’). The method ‘{3 h}+*T*_phys_’ allows us to investigate the potential bias that could occur in dose estimates calculated in the treatment planning of the IAEA ^188^Re-AHDD-Lipiodol clinical trial [13, 24] which assumed that the biological clearance of ^188^Re-HDD-Lipiodol by tumor and other organs was negligible, while the case ‘{3 h}+*T*_eff_’ was selected to investigate the accuracy of the simplified method based on a single time-point scan which we propose in this work.

### Statistical analysis

The Mann-Whitney-Wilcoxon test [42, 43] was applied to determine if there was any statistical difference between the effective half-lives (for each organ of interest) derived from imaging data at {3 h, 24 h}, {3 h, 48 h}, and {24 h, 48 h}.

The relative differences of TIACs ($\overset{\sim }{a_X}$) derived from data acquired at {3 h, 24 h}, {3 h, 48 h}, {24 h, 48 h}, ‘{3 h} + *T*_phys_’, and ‘{3 h} + *T*_eff_’ with respect to those from data at {3 h, 24 h, 48 h} were presented in the form of box-plots. In these plots, the central mark in each box indicates the median of the distribution, and the bottom and top borders of the box indicate the 25th and 75th percentiles, respectively. The whiskers extend to the most extreme data-points that were within 1.5 times the inter-quartile range both above and below the box borders. Measurements that were beyond the whiskers limits were considered outliers.

The Mann-Whitney-Wilcoxon test was also applied to determine the significance of the differences observed between the TIACs derived from the reference protocol and those derived from imaging data at {3 h, 24 h}, {3 h, 48 h}, {24 h, 48 h}, ‘{3 h} + *T*_*phys*_’ and ‘{3 h} + *T*_*eff*_’.

## Results

### Biodistribution of ^188^Re-AHDD-Lipiodol

**Fig. 2:**
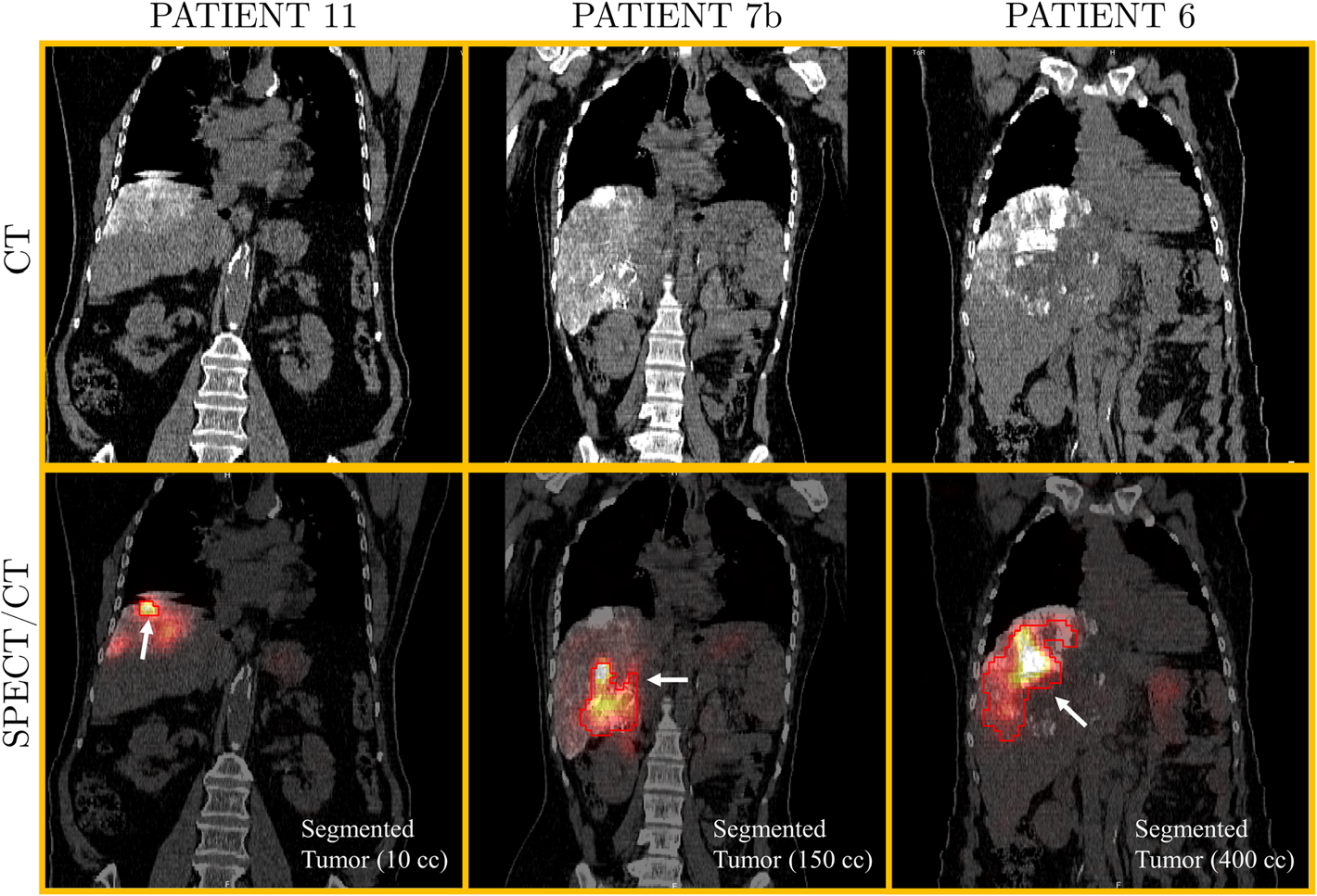
CT and SPECT/CT coronal views of patient no. 11, 7b, and 6 showing the distribution of ^188^Re-HDD-Lipiodol in liver tissue at 3 h after administration of the radiopharmaceutical

Figure 2 (top) shows CT coronal slices of the same three patients of Fig. 1, acquired at *t*_1_= 3 h after administration of ^188^Re-AHDD-Lipiodol. Thanks to the high-density of Lipiodol (which contains iodine), its deposition in liver tissue is visible on the CT image. The corresponding fused SPECT/CT coronal slices of these patients are shown on Fig. 2 (bottom) together with the segmented tumor boundaries. Large non-uniformities of ^188^Re-AHDD-Lipiodol deposition within the liver are visible on the SPECT/CT data. Faint accumulation of ^188^Re-AHDD-Lipiodol in stomach and normal liver can also be observed (Fig. 2).

### Pharmacokinetics of ^188^Re-AHDD-Lipiodol

**Fig. 3:**
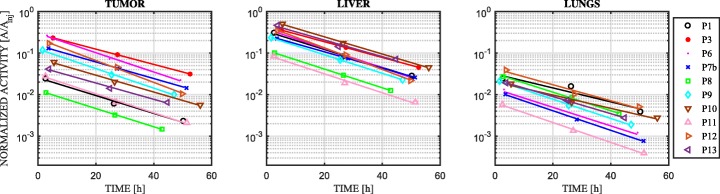
Normalized time-activity curves of tumor, liver and lungs for patients that were scanned three times after ^188^Re-HDD-Lipiodol administration

The TACs for the tumor, liver, and lungs are plotted in Fig. 3. For comparison purposes, the curves were normalized by the injected activity. To illustrate the quality of the mono-exponential fit, only curves derived from patient data that contained three imaging time-points are shown. The high coefficients of determination of the fit (*R*^2^) proved that the mono-exponential function fit the data well in all investigated regions of interest (both tumor and organs at risk). The minimum *R*^2^ values were 0.989, 0.984, and 0.916 for the tumor, liver, and lungs, respectively.

These TACs illustrate two important features. Firstly, their slopes are similar to each other, indicating that the effective half-lives of ^188^Re-AHDD-Lipiodol in the tumor, liver, and lungs are similar. Secondly, since the TACs were normalized by the injected activity, the broad variability in these normalized curves for tumors implies that the corresponding TIACs (i.e., the area under these curves) also varied largely across patients.

**Fig. 4:**
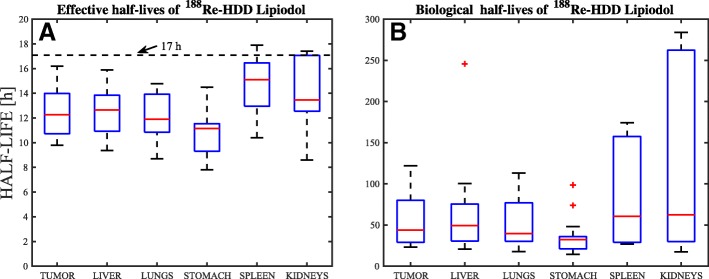
Distributions of the effective (**a**) and biological (**b**) half-lives of ^188^Re-HDD-Lipiodol in the tumor, liver, lungs, stomach, spleen, and kidneys. The horizontal dashed-line (sub-A) indicates the physical half-life of ^188^Re (i.e., 17 h)

The distributions of the effective half-lives of ^188^Re-AHDD-Lipiodol in tumor and organs at risk are depicted in Fig. 4a in the form of box-plots. On average (± standard deviation across all patients), the effective half-lives of this radiopharmaceutical were equal to 12.5 ± 1.9h, 12.6 ± 1.7h, 12.0 ± 1.9h, and 12.8±2.5 h for the tumor, liver, lungs, and kidneys, respectively. The stomach showed the fastest clearance among all the investigated organs, with an effective half-life of 10.9 ± 1.9 h whereas spleen showed the slowest washout of ^188^Re-AHDD-Lipiodol, with effective half-life of 14.7 ± 2.3h.

The effective half-lives of the tumor and organs at risk were significantly lower than the physical half-life of ^188^Re, indicating that there is a measurable biological washout of the radiopharmaceutical. Using Eq. 2, the biological half-lives were estimated for each tissue of interest, and their distributions are shown in the box-plots of Fig. 4b. On average, the biological half-lives of ^188^Re-AHDD-Lipiodol in tumor, liver and lungs were 50.7±29.7 h, 49.8±25.1 h, and 49.4±28.4 h, respectively. For spleen and kidneys, the biological half-lives were 83.0 ± 55 h and 81.9 ± 83 h, respectively. Lastly, the biological half-life of ^188^Re-AHDD-Lipiodol in stomach was 28.9 ± 9.5 h.

### Organ dosimetry

Table 2 reports the absorbed doses per injected activity (referred to as “normalized doses”) for each tissue of interest and for each patient, their average values over the entire patient dataset, and the un-normalized average values. On average, tumor normalized doses (23.5 ± 40.8 mGy/MBq) were approximately 10 times higher than those in the entire liver (2.12 ± 1.78 mGy/MBq). Other organs at risk received much lower doses than the tumor and liver, which was expected, considering the low accumulation of the radiopharmaceutical observed in planar and SPECT images.

**Table 2:** Normalized absorbed doses per injected activity (in mGy/MBq) in tumors and organs at risk for each patient, and their average over the entire dataset. The standard deviation (± SD) and the minimum and maximum normalized doses are also reported. Additionally, the absolute mean (± SD), minimum, and maximum absorbed doses are reported

Patient no.	Tumor	Liver	Lungs	Stomach	Spleen	Kidneys
1	13.92	2.41	0.09	0.58	0.88	0.36
2	5.71	1.37	0.20	0.63	0.45	0.31
3	2.56	0.99	N/A	0.26	0.36	N/A
4	8.99	1.19	0.11	0.32	0.14	0.25
5	31.94	2.39	0.16	0.20	0.12	0.10
6	5.46	1.24	0.06	0.29	0.25	0.22
7a	3.65	1.98	0.07	0.13	0.53	0.45
7b	9.17	2.31	0.09	0.05	0.33	0.24
8	9.99	0.86	0.11	0.31	0.46	0.18
9	14.75	1.03	0.08	0.53	0.15	0.13
10	53.83	3.78	0.13	0.45	0.68	0.48
11	2.32	0.48	0.02	0.77	0.09	N/A
12	4.76	1.69	0.17	0.20	0.73	0.17
13	162.49	7.82	0.13	0.16	0.21	N/A
Mean ± SD	23.5 ± 40.8	2.12 ± 1.78	0.11 ± 0.05	0.35 ± 0.20	0.38 ± 0.24	0.26 ± 0.12
Min	2.32	0.48	0.02	0.05	0.09	0.10
Max	162.49	3.78	0.20	0.77	0.88	0.48
Absolute mean [Gy]	50.4 ± 66.4	6.1 ± 4.0	0.3 ± 0.2	1.1 ± 0.9	1.4 ± 1.1	0.9 ± 0.7
Absolute min [Gy]	4.0	0.8	0.03	0.3	0.2	0.2
Absolute max [Gy]	266.0	12.8	1.1	3.8	3.0	2.4

An important observation which can be made from the analysis of the results presented in Table 2 is the large inter-patient variability of normalized doses, resulting in the very large standard deviations. For instance, the tumor normalized doses ranged from 2.32 to 162.49 mGy/MBq. Similarly, the liver doses ranged from 0.48 to 3.78 mGy/MBq.

### Effect of scan frequency and timing on the estimated pharmacokinetics and time-integrated activity coefficients

**Fig. 5:**
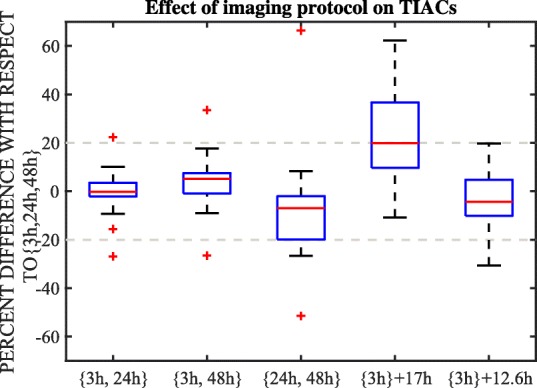
Distribution of the percent difference of the time-integrated activity coefficients determined using imaging data at {3 h, 24 h}, {3 h, 48 h}, {24 h, 48 h}, {3 h} + physical half-life (i.e., 17 h), and {3 h} + effective half-life (i.e., 12.6 h) with respect to those at {3 h, 24 h, 48 h} (reference dataset). The time-integrated activity coefficients presented in this figure correspond to the tumor, liver, and lungs only

Figure 5 shows the percent-difference distribution (in the form of box-plots) of the TIACs estimated with the {3 h, 24 h}, {3 h, 48 h}, {24 h, 48 h}, {3 h}+*T*_phys_ and {3 h}+*T*_eff_ datasets with respect to the reference values (estimated with the imaging data at {3 h, 24 h, 48 h}). This figure implies that using only imaging data acquired at {3 h, 24 h} results in TIACs very similar to those derived from the complete dataset. In particular, the percent differences were most of the times within 6%, and always less than 10% (without including the two outliers that yielded differences > 20%). The use of imaging data at {3 h, 48 h} or at {24 h, 48 h} increased the percent differences of the estimated TIACs, but these differences were still within ± 20%. The TIACs from data acquired at {3 h, 24 h}, {3 h, 48 h}, and {24 h, 48 h} were not significantly different from the reference TIACs (*p* values = 1.0, 0.51, and 0.47, respectively).

The use of one single imaging point (at {3 h}) combined with the physical half-life of ^188^Re resulted in the large differences in TIACs with respect to the reference values. More than half of the estimated TIACs were > 20% higher than the reference values, which translates into similar > 20% overestimation of the absorbed dose for these cases (as dose is proportional to TIACs). The Mann-Whitney-Wilcoxon test indicated, however, that these differences were not statistically significant (*p* value = 0.14). Interestingly, if one single imaging point (at {3 h}) is combined with the effective half-life of ^188^Re-AHDD-Lipiodol determined in our study (reference value), the majority of estimated TIACs would still be within ± 20% of the reference values for most of the data. Furthermore, no significant difference between this protocol and the reference protocol was found (Mann-Whitney-Wilcoxon, *p* value = 0.73).

## Discussion

### Biodistribution and pharmacokinetics of ^188^Re-AHDD-Lipiodol

Figure 1 shows that ^188^Re-AHDD-Lipiodol is mostly accumulated in the liver, but a non-negligible amount of this radiotracer is also seen in other organs such as the lungs, the stomach, the spleen, the salivary glands, the thyroid, the kidneys, and the urinary bladder. Lipiodol is a high-viscosity compound derived from poppy-seed oil that does not necessarily behave like a physical embolization agent. Previous in vitro and in vivo studies have shown that Lipiodol can be selectively retained for a long time in tumor tissue after intra-arterial administration [44–46]. Originally, Lipiodol was labelled with ^131^I for liver radioembolization by replacing cold iodine (which is present in Lipiodol) with radioactive iodine [47].

However, this approach is not valid for labelling with ^188^Re. In this case, labelling is achieved by preparing a strongly lipophilic Re-complex which is then solubilized in strongly hydrophobic Lipiodol [48]. Therefore, the stability of ^188^Re-Lipiodol complexes in vivo is not necessarily the same as that of ^131^I-Lipiodol or Lipiodol itself.

In most of the patients, we have observed that the highest signal of ^188^Re-AHDD-Lipiodol correlates well with the approximate location of tumors reported by the physicians (Fig. 2). However, we also observed non-negligible amounts of ^188^Re-AHDD-Lipiodol outside the tumor area. We believe this effect might be caused by (a) the hepatic artery supplying some blood to regions of the healthy liver and (b) the fact that Lipiodol does not behave exactly like a physical embolization agent such as glass or resin microspheres.

Lambert et al. [30] reported the biological clearance of ^188^Re-HDD-Lipiodol from tissues based on (a) the observed urinary excretion of free perrhenate activity and (b) the measured WB effective half-life (using imaging) of 14.3 h which was shorter than the ^188^Re physical half-life. The results from our study are in agreement with those from Lambert as our post-treatment patient images showed uptake of ^188^Re in thyroid, salivary glands, and urinary bladder (Fig. 1), strongly suggesting that free ^188^Re is released from the labelled complex with a measured effective half-life of 12.6 h in liver tissue. Another evidence supporting this claim is poor correlation between the Lipiodol deposition (as seen on CT) and the ^188^Re SPECT activity distribution observed in some patients (Fig. 2, patient 7b and 6). Our results also agree with pre-clinical measurements of ^188^Re-HDD-Lipiodol in rabbit liver tumors, where the measured effective half-life was approximately 12 h [25]. A very comprehensive description of the chemistry of Lipiodol labelling with ^188^Re for radioembolization has been recently published by Lepareur and Garin [49].

Despite the limited in vivo stability of ^188^Re-AHDD-Lipiodol, the IAEA clinical study [13] showed that this radiopharmaceutical is a safe and cost-effective treatment for hepatocellular carcinoma, especially for developing countries where the high-cost of radioembolization therapy with ^90^Y-microspheres often impedes its use. Alternatively, new and more stable radio-conjugates labelled with ^188^Re have been proposed as promising candidates for radioembolization therapy, such as ^188^Re-SSS-Lipiodol [50] or ^188^ReN-DEDC-Lipiodol [51]. Another candidate for radioembolization is ^188^Re-Human Serum Albumin, which has been shown to be stable in vivo, safe and effective [2, 52]. Most recently, bio-degradable ^188^Re-microspheres [53] and starch-based ^188^Re-microparticles [54] have been investigated in pre-clinical studies as a new generation of carriers for radioembolization.

### Organ dosimetry

The results reported in Table 2 illustrate the very large inter-patient variability of normalized doses absorbed by the tumor (ranging from 2.32 to 162.49 mGy/MBq) and the organs at risk. Since the pharmacokinetics (i.e., effective half-life) of ^188^Re-AHDD-Lipiodol was very consistent across patients (slopes in Fig. 3 and first column of Table 3), the large inter-patient variability observed in tumor doses was mostly due to the differences in ^188^Re-AHDD-Lipiodol uptake. A closer look at Fig. 3 (tumor) shows that this uptake ranged from 1% (patient 8) to almost 30% (patients 6 and 3) of the injected activity. These results highlight the importance of performing post-treatment personalized dose calculations as the accumulation of ^188^Re-AHDD-Lipiodol in tissue per injected activity strongly varies between patients. Such accurate and personalized dose calculations would be essential for the investigation of potential correlations between absorbed doses in tumor and organs at risk, and patient’s response to treatment, as well as healthy tissue toxicities. It is important to note that, despite the large inter-patient variability of normalized dose, the absolute doses cumulated in the liver and lungs (the two most critical organs at risk) were at most 12.8 Gy and 2.5 Gy, respectively (Table 2). These values are well below these organs maximum tolerated doses of 30 Gy (liver) [26] and 12 Gy (lungs) [27], respectively. Although the doses to healthy liver tissue were not estimated in our study because the pre-calculated S-values were only available for the entire organ, these doses are expected to be lower than those in the entire liver because the healthy liver tissue excludes the contribution from the tumor dose.

**Table 3:** Effective half-lives of tumors and organs at risk determined using patient imaging data at {3 h, 24 h, 48 h},{3 h, 24 h}, {3 h, 48 h}, and {24 h, 48 h}. The data is compared to an effective half-life of 17 h (which would assume that AHDD-Lipiodol biological half-life is infinity, as in Zanzonico et al 2008 [29]). The data are presented as the average effective half-life across all patients ± SD in hours

	{3 h, 24 h, 48 h} (reference value)	{3 h, 24 h}	{3 h, 48 h}	{24 h, 48 h}	{3 h}
Tumor	12.5 ± 1.9	12.0 ± 1.4	13.8 ± 1.4	16.2 ± 2.0	17
Liver	12.6 ± 1.7	12.4 ± 1.1	13.9 ± 1.2	16.0 ± 1.9	17
Lungs	12.0 ± 1.9	12.1 ± 1.3	13.6 ± 1.2	15.6 ± 2.4	17
Stomach	10.9 ± 1.9	11.4 ± 1.5	12.8 ± 1.3	14.9 ± 2.3	17
Kidneys	12.8 ± 2.5	13.8 ± 1.8	14.5 ± 1.2	15.5 ± 2.8	17
Spleen	14.7 ± 2.3	14.1 ± 1.5	15.1 ± 1.8	16.4 ± 1.8	17

The range of tumor doses delivered in our study (50.4 ± 66.4 Gy) was very similar to those estimated in the IAEA collaboration (66.4 ± 59.9 Gy) [13, 24]. These tumor doses were much lower than the typical doses delivered during ^131^I (248 ± 176 Gy) [16] or ^90^Y (183 ± 101 Gy) [55] radioembolization treatments. The main factor which limits radiation doses in ^188^Re-Lipiodol treatments is the low effective half-life of this radiopharmaceutical compared to ^90^Y microspheres or ^131^I-Lipiodol. To compensate for this effect, the ^188^Re administered activity would need to be increased which might be challenging, as it would require that much larger volumes to be injected. This, however, was impossible in our study due to the limited labelling efficiency of AHDD Lipiodol kits, as well as the low yield of the ^188^W-^188^Re generator at the time of its use.

The proposed tumor segmentation method, which was based on the application of a fixed threshold to the SPECT image, imposed some limitations on the calculation of tumor dose. Our method was based on the assumption that ^188^Re SPECT signal correlates well to the physical tumor boundaries, which might not always be true (Fig. 2). To improve the accuracy of tumor dose estimations, the tumor boundaries should be defined on the ceCT images. These images can be registered with SPECT/CT data, and the segmented volume can be subsequently applied to the SPECT image to determine the activity (and therefore, the dose) in the tumor. However, processing of such ceCT images was not available in our study.

One aspect that is worth discussing concerns the accuracy of the dose estimates using the presented imaging and dosimetry method. Without the knowledge of the true activity distribution in patients, we can only estimate the accuracy of the applied method. Assuming that the pharmacokinetics of the radiopharmaceutical and the calculation of the TIACs had no errors (which is unrealistic), then the accuracy of dose estimates only relies on the accuracy of our determination of tumor and organ activities using SPECT images.

Our phantom studies [19] have shown that, when using the clinical reconstruction method described in section 'Quantitative SPECT for absolute TACs', the errors in the measurements of activities in SPECT images were approximately less than 25% for objects with volumes > 20 mL. These errors are mostly related to segmentation inaccuracies, caused by partial volume effect. As these effects decrease with increasing volume of the segmented object, we expect the errors in organs’ activities to be smaller than 25%, as all the investigated organs have volumes much larger than 20 mL. This is not the case for small tumors, where we expect radiation doses to be underestimated by approximately 25–40% due to the strong influence of partial volume effects. We expect that the tumor dosimetry error of patient 13 is even higher than 40% due to its really small size (2.3 mL), very close to the resolution limit of the SPECT system.

Another limitation of the applied dosimetry method that might affect accuracy of estimated doses is that the organ-level dose estimates were based on pre-calculated S-values from standard male/female phantoms, whose anatomy (organ sizes and separation between organs) might differ from the real patients. To minimize this problem, the S-factors were re-scaled to account for patient-specific organ volumes. Such correction does not account for the cross-dose contributions due to differences between the real patient’s anatomy and the standard phantom. For ^188^Re, however, the cross-doses due to energy deposition of *γ*-emissions are negligible compared to the self-doses (which are caused by local deposition of energy by *β*^−^-emissions).

The clinical team at Kovai Medical Center and Hospital is now conducting a follow-up study of the patients treated in this study. In this context, our future work will be the investigation of the relationship between average absorbed dose and tumor response, and long-term toxicities for these patients. It is important to note that the current dosimetry was performed at the organ-level. That is, only the dose averaged over the entire organ was reported. Since liver toxicities are more likely to be related to healthy liver doses (and not to entire liver doses), our future analysis will involve determination of the normal liver doses, as well as doses of tumors and other organs at risk, using a voxel-level dosimetry approach [56]. Such dosimetry will allow us to explore the potential correlations between parameters of the 3-dimensional dose-distribution (minimum and maximum dose in tumor, dose-volume histograms, etc.) and patient response as these parameters might constitute better predictors of deterministic effects than the average organ doses [20, 57]. Furthermore, voxel-level dosimetry will also improve the accuracy of tumor doses as contrary to OLINDA, and it would not use the spherical tumor model. This may especially benefit dose estimates for small tumors with irregular shapes.

### Effect of scan frequency and timing on estimated pharmacokinetics and time-integrated activity coefficients

The comparison of the pharmacokinetics and TIACs estimated based on different subsets of the imaging data was presented in Table 3 and Fig. 5. The data in Table 3 show that the effective half-life of ^188^Re-AHDD-Lipiodol was significantly shorter during the first 24 h post-administration than during the 24–48-h interval. We explain this finding with the following hypothesis: during the first 24 h, the measured change of ^188^Re activity in tissue is mostly due to the chemical separation of this radionuclide from Lipiodol (i.e., measured effective half-life is ~ 12–13 h), while during the 24–48-h interval, the measured activity in tissue (liver, tumor and lungs) is mostly due to ^188^Re that remained bounded to Lipiodol, and hence, the change in activity is dominated by ^188^Re physical decay, resulting in a measured effective half-life of approximately 16 h ≈*T*_phys_.

This observation indicates that the reference clinical protocol (three imaging points) cannot model the true pharmacokinetics, as with only three imaging points it is impossible to fit a bi-exponential function. In order to accurately model the bi-exponential function, patients would need to be scanned at least four or five times. Although such protocol would increase the accuracy of time-activity curve determination, its effect on the overall TIAC is expected to be low for ^188^Re-AHDD-Lipiodol radioembolization. Indeed, the data presented in Table 3 shows that the reference protocol under-estimates the effective half-life in liver/tumor by approximately 20% in the 24–48-h interval, whereas it is in good agreement with the half-life measured in the 3–24-h interval. As a result, the time-integrated activity coefficients derived from the reference protocol would only be 6% lower than those assuming a bi-exponential clearance.

The imaging protocol applied during the IAEA study had limitations which could have led to sub-optimal treatment planning. Firstly, the protocol was based on a single ^188^Re WB planar image which must have had limited quantitative accuracy [58]. Secondly, the protocol relied on the assumption that biological clearance of ^188^Re-HDD Lipiodol by tumor and other organs was very slow. Therefore, the effective half-life of this compound in tissue was modelled by the physical half-life of ^188^Re. This assumption, which was based on the ^131^I-Lipiodol studies [59], was not verified during the IAEA clinical study because post-treatment imaging was not performed.

Figure 5 illustrates the potential bias in the estimated time-integrated activity coefficients (and therefore, the absorbed doses) of the IAEA clinical study. Our data suggest that, on average, the doses might have been overestimated by 20% (as compared to the reference values with the measured effective half-life). It is important to mention that the IAEA dosimetry protocol was intended only for treatment planning, and hence, the assumption of no biological clearance was rather a conservative approach to ensure that the predicted injected activity would yield doses for organs at risk below the tolerance limits.

The small variability of the effective half-life of ^188^Re-AHDD-Lipiodol in tumor, liver, and lungs, derived from patient images allowed us to explore the accuracy of determination of the TIACs using the simplified protocol ‘{3 h}+*T*_eff_’ as compared to the reference values. Figure 5 shows that if one single SPECT/CT is performed and the obtained tumors/organ activities are combined with the average effective half-life of ^188^Re-AHDD-Lipiodol, the estimated TIACs deviate by less than 20% from the reference values for most of the cases. Such acquisition protocol would greatly simplify dose calculations, increasing the likelihood of them being implemented routinely in every nuclear medicine department performing ^188^Re-AHDD-Lipiodol radioembolization treatment. Most importantly, it would decrease the patient imaging time (less scans), reduce the burden on patients, and potentially decrease the hospitalization time while maintaining acceptable accuracy of dose estimates. For these reasons, we would recommend this imaging-dose protocol for: (a) centers that are implementing ^188^Re-AHDD-Lipiodol radioembolization and might not have enough resources and tools to perform complex dosimetry or (b) for patients that cannot be imaged multiple times. Please note that this protocol is only applicable to therapies with AHDD-Lipiodol as any other radioconjugate (such as N-DEDC-Lipiodol or SSS-Lipiodol) might show different pharmacokinetic behavior in vivo. The single SPECT/CT protocol is described step-by-step in the Additional file 1.

## Conclusions

In the present study, we performed personalized dosimetry for 13 patients undergoing ^188^Re-AHDD-Lipiodol radioembolization using quantitative post-treatment imaging. The analysis of quantitative SPECT and whole-body data showed the presence of ^188^Re in organs other than the liver which was probably due to the chemical separation and subsequent release of the free radionuclide from Lipiodol. This effect resulted in an effective half-life of ^188^Re-AHDD-Lipiodol equal to 12.6 ± 1.7 h in liver/tumor tissue.

The large inter-patient variability of normalized doses delivered to the tumors (23.5 ± 40.8 mGy/MBq), livers (2.12 ± 1.78 mGy/MBq), and lungs (0.27 ± 0.13 mGy/MBq) strongly corroborates the importance of performing patient-specific dose calculations.

Based on the results of this study, we propose a simple dosimetry protocol. It consists of a single SPECT/CT imaging study combined with the population-based ^188^Re-AHDD-Lipiodol effective half-life. Employing this protocol to our patient datasets resulted in TIACs estimates (and hence, the doses) mostly within ± 20% from the reference TIACs (estimated using three whole-body images and one SPECT/CT for each patient).

## Additional file

Additional file 1: A practical image-based dosimetry protocol for ^188^Re-AHDD-Lipiodol (PDF 1040 kb)

## Abbreviations

^188^Re-AHDD-Lipiodol: Rhenium-188-labelled diacetylated 4-hexadecyl-1,2,9,9-tetramethyl-4,7-diaza-1,10-decanethiol-Lipiodol
ceCT: Contrast-enhanced CT
IAEA: International Atomic Energy Agency
OAR: Organ at risk
OSEM: Ordered-subset expectation maximization
ROI: Region of interest
TAC: Time-activity curve
TEW: Triple-energy window
TIAC: Time-integrated activity coefficient
WB: Whole body
