# Supplementary material for: Biodistribution, pharmacokinetics, and organ-level dosimetry for 188Re-AHDD-Lipiodol radioembolization based on quantitative post-treatment SPECT/CT scans
Source: EJNMMI Phys. 2018 Dec 7;5:30. doi: 10.1186/s40658-018-0227-6 (PMC6283804; doi:10.1186/s40658-018-0227-6)
Supplement: Supplementary file 1 — A practical image-based dosimetry protocol for 188Re-AHDD-Lipiodol (PDF 1040 kb) [file 40658_2018_227_MOESM1_ESM.pdf]

SUPPLEMENTARY MATERIAL

Biodistribution, Pharmacokinetics and Organ Level Dosimetry for ^{188}Re AHDD-Lipiodol Radioembolization Based on Quantitative Post-treatment SPECT/CT Scans

Pedro L. Esquinas[1,2], Ajit Shinto[3] ,K. K. Kamaleshwaran[3], Jephy Joseph[3], and Anna Celler[1,2]

[1]*Department of Radiology, University of British Columbia, Vancouver, BC, Canada*

[2]*Medical Imaging Research Group, Vancouver, BC, Canada*

[3]*Department of Nuclear Medicine, Kovai Medical Center and Hospital, Coimbatore, Tamil Nadu, India*

Corresponding Author

Name: Dr. Pedro Luis Esquinas

Address: Medical Imaging Research Group,

367 – 828 West 10th Avenue,

Vancouver, BC

Canada V5Z 1L8

Tel: (+1) 604 875-5252

Email: esquinas@mail.ubc.ca

A practical image-based dosimetry protocol for ^{188}Re-AHDD-Lipiodol

1.1 Image acquisition and quantitative image reconstruction

- Acquire SPECT tomographic data at 3-hours after administration of ^{188}Re-AHDD-Lipiodol. The acquisition parameters are as follows:
  - High-energy collimator (preferred); Medium-energy collimator is acceptable but the study might suffer from high dead-time losses. Low-energy high-resolution collimator is not recommended for imaging ^{188}Re (1).
  - At least 32 projections of 20 seconds duration.
  - Photopeak window centered at 155 keV (20% width) with upper and lower scatter windows around the photopeak (each having 5% to 10% width).
- Acquire low dose CT and generate the attenuation map for attenuation correction.
- Determine the detected count-rate in the photopeak window at each projection image and average it over all projections. This quantity ($\overline{CR}_{photo}$) will be used to estimate the dead-time correction factor, if applicable.
- Using the average photopeak count-rate, determine the corresponding dead-time correction factor ($DTCF(\overline{CR}_{photo})$) from the tabulated data shown in Table 1S. Please note that these dead-time correction factors were determined from a phantom calibration experiment using SymbiaT SPECT/CT (Siemens Medical,

Germany) and may not be applicable to other SPECT systems. The details of this method can be found in (2).

- Reconstruct the projection data using OSEM with 8 subsets, 12 iterations and corrections for attenuation (using attenuation map), scatter (using the triple-energy window method), and resolution loss.
- Correct the voxels in the reconstructed image (R) with the tabulated dead-time correction factor (if needed). The resultant image ($R_{corrected}$) represents the 3-dimensional distribution of counts corrected for attenuation, scatter, resolution loss and dead-time:

$$R_{corrected} = R \times DTCF(\overline{CR}_{photo})$$

- Apply the camera calibration factor (CF) to the reconstructed image ($R_{corrected}$) to convert counts in each voxel into units of activity. A method to determine CF is described in (2). The resultant image (A) represents the 3-dimensional distribution of activity within the patient:

$$A = R_{corrected} \times CF$$

1.2 Image segmentation and time-integrated activity coefficient determination

- Register the reconstructed SPECT image is with its corresponding CT. If the system is a hybrid SPECT/CT, this step may not be required as the images typically are already registered. However, it is very important to verify that this registration is accurate as there could have been misalignment due to patient motion or due to hardware/software errors.

- Segment the liver and the lungs (i.e., the critical organs at risk) using the physical boundaries on the CT image. Please note that the presented simplified protocol for dosimetry is only acceptable for these organs as we have shown that the variability of the effective half-life for these organs across patients was relatively small.
- Copy the segmented liver and lung volumes into the SPECT image and calculate the activity in the liver (A_{LIVER}) and the lungs (A_{LUNGS}) by summing the voxel activities within these volumes.
- Draw a rough volume around the liver segment where the tumor is located and segment it using a threshold value that yields a volume equal to the tumor volume (known from ceCT pre-treatment imaging). Note that this method relies on the assumption that ^{188}Re-AHDD-Lipiodol deposition in liver correlates well with the tumor location. This assumption should be, at least, verified by visually comparing the pre-treatment ceCT image and the post-treatment SPECT/CT. If the correlation between the tumor and ^{188}Re-AHDD-Lipiodol deposition is poor, it is recommended to segment the tumor on the ceCT image and register the ceCT image (including the segmented tumor boundaries) to the SPECT/CT data.
- Calculate the activity within the segmented tumor volume (A_{TUMOR}).
- Estimate the time-integrated activity in the tumor/organ of interest ($\tilde{A}(r_S, T_D)$, with $T_D = \infty$) using the following expression:

$$\tilde{A}(r_S, T_D) = \int_0^{\mathrm{T_D}} A(r_S, t)dt$$

$$\approx t \times \frac{A_S}{2} + A_S\left(1 - e^{-\frac{ln2}{T_{eff}}(48-t)}\right)\left(\frac{T_{eff}}{ln2}\right) + A_S e^{-\frac{ln2}{T_{eff}}(48-t)}\left(\frac{T_{phys}}{ln2}\right)$$

where the label S represents $LIVER, LUNGS$ or $TUMOR$; t is the SPECT acquisition time (in hours, after administration of ^{188}Re-AHDD-Lipiodol); $T_{eff} = 12.6$ h represents the average effective half-life of ^{188}Re-AHDD-Lipiodol in tissue and $T_{phys} = 17$ h is the physical half-life of ^{188}Re.

- Calculate the time-integrated activity coefficient of the organ/tumor of interest ($\tilde{a}(r_S, T_D)$):

$$\tilde{a}(r_S, T_D) = \frac{\tilde{A}(r_S, T_D)}{A_0}$$

where A_0 represents the injected activity at time of injection, in MBq.

1.3 Organ-level dosimetry calculations

- Determine the normalized dose per injected activity (in mGy/MBq) absorbed by liver and lungs by multiplying the residence times with the corresponding pre-calculated S-factors. Tabulated S-factors can be found in available internal dosimetry software tools such as IDAC 1.0/2.0 (3) and OLINDA/EXM 1.1 (4). If the option is available, re-scale the S-factor to account for the patient-specific organ volume (as determined from CT). Table 2S contains the volume-dependent liver and lungs S-Factors for ^{188}Re, obtained from IDAC 1.0.

- Determine the normalized dose per injected activity absorbed by the tumor by multiplying the tumor time-integrated activity coefficient by a spherical-volume-dependent S-factor. Table 2S reports ^{188}Re S-Factors for spheres of different volumes.
- The total dose absorbed by the liver, the lungs and the tumor can finally be obtained by multiplying the normalized doses by the injected activity.

Table 1S Tabulated deadtime correction factors as a function of the count-rate in the ^{188}Re photopeak window (155 keV, 20% width) for medium energy and high energy collimators.

MEDIUM ENERGY		HIGH ENERGY	
Photopeak count-rate (kcps)	Correction factor	Photopeak count-rate (kcps)	Correction factor
0.00	1.000	0.00	1.000
1.21	1.007	1.73	1.001
5.41	1.017	4.43	1.002
9.48	1.030	7.11	1.006
13.40	1.046	9.76	1.010
17.12	1.067	12.38	1.016
20.63	1.091	14.94	1.023
23.88	1.119	17.43	1.032
26.87	1.151	19.86	1.042
29.57	1.188	22.21	1.053
31.97	1.229	24.46	1.067
34.07	1.276	26.62	1.082
35.86	1.328	28.67	1.099
37.36	1.385	30.61	1.117
38.56	1.448	32.43	1.137
39.49	1.518	34.13	1.160
40.16	1.594	35.70	1.184
40.59	1.677	37.14	1.210
40.80	1.767	38.46	1.239

Table 2S Tabulated ^{188}Re S-Factors for Liver and Lungs self-organ dose as a function of the organ mass. The S-Factors are calculated for time-integrated activity coefficient = 1 MBq h/MBq. To obtain the absorbed dose per injected activity, multiply these tabulated factors by the organ time-integrated activity coefficient. These S-Factors were obtained from IDAC 1.0.

LIVER		LUNGS	
Mass (g)	S_{LIVER} (mGy/MBq) ($\times 10^{-1}$)	Mass (g)	S_{LUNGS} (mGy/MBq) ($\times 10^{-1}$)
1000	4.54	500	8.89
1100	4.13	600	7.42
1200	3.79	700	6.36
1300	3.50	800	5.57
1400	3.25	900	4.96
1500	3.03	1000	4.51
1600	2.84	1100	4.10
1700	2.68	1200	3.76
1800	2.53	1300	3.47
1900	2.40	1400	3.23
2000	2.28	1500	3.01

Table 3S Tabulated ^{188}Re S-Factors for water spheres of increasing mass. The S-Factors are calculated for time-integrated activity coefficient = 1 MBq h/MBq. To obtain the absorbed dose per injected activity, multiply these tabulated factors by the tumor's time-integrated activity coefficient. These S-Factors were obtained from IDAC 1.0.

SPHERE MODEL	
Mass (g)	S-Factor (mGy/MBq)
10	4.97×10^{1}
20	2.08×10^{1}
40	1.05×10^{1}
60	7.08×10^{0}
80	5.34×10^{0}
100	4.29×10^{0}
300	1.46×10^{0}
400	1.10×10^{0}
500	8.86×10^{-1}
600	7.41×10^{-1}
1000	4.49×10^{-1}

References

1. Uribe CF, Esquinas PL, Gonzalez M, Celler A. Characteristics of Bremsstrahlung emissions of 177Lu, 188Re, and 90Y for SPECT/CT quantification in radionuclide therapy. Phys Medica. Associazione Italiana di Fisica Medica; 2016;32(5):691–700.
2. Esquinas PL, Uribe CF, Gonzalez M, Rodríguez-Rodríguez C, Häfeli UO, Celler A. Accuracy of Rhenium-188 SPECT/CT activity quantification for applications in radionuclide therapy using clinical reconstruction methods. Phys Med Biol. 2017;62(16):6379–96.
3. Andersson M, Johansson L, Minarik D, Mattsson S, Leide-Svegborn S. An Internal Radiation Dosimetry Computer Program , IDAC 2 .0, for Estimation of Patient Doses From Radiopharmaceuticals. Radiat Prot Dosimetry. 2013;162(3):299–305.
4. Stabin MG, Sparks RB, Crowe E. OLINDA/EXM: The Second Generation Personal Computer Software for Internal Dose Assessment in Nuclear Medicine. J Nucl Med. 2005;46:1023–7.
